# The Impact of Intermittent Hypoxia-Hyperoxia Therapy on Metabolism and Respiratory System in Obese Patients as Part of Comprehensive Medical Rehabilitation

**DOI:** 10.7759/cureus.71501

**Published:** 2024-10-14

**Authors:** Andreea-Bianca Uzun, Madalina Iliescu, Liliana-Elena Stanciu, Andreea-Dalila Nedelcu, Adina Petcu, Marius N Popescu, Cristina Beiu, Lucian Cristian Petcu, Doina-Ecaterina Tofolean

**Affiliations:** 1 Department of Physical Medicine and Rehabilitation, Ovidius University of Constanta - Faculty of Medicine, Constanta, ROU; 2 Department of Physical and Rehabilitation Medicine, Ovidius Doctoral School of Medicine, Constanta, ROU; 3 Department of Physical and Rehabilitation Medicine, Balneal and Rehabilitation Sanatorium of Techirghiol, Constanta, ROU; 4 Department of Physical Medicine and Rehabilitation, Ovidius Doctoral School of Medicine, Constanta, ROU; 5 Department of Physical Medicine and Rehabilitation, Balneal and Rehabilitation Sanatorium of Techirghiol, Constanta, ROU; 6 Department of Pharmaceutical Sciences, Ovidius University of Constanta - Faculty of Pharmacy, Constanta, ROU; 7 Department of Radiotherapy, Ovidius Clinical Hospital, Constanta, ROU; 8 Department of Physical Medicine and Rehabilitation, Carol Davila University of Medicine and Pharmacy, Bucharest, ROU; 9 Department of Oncologic Dermatology, Elias Emergency University Hospital, Carol Davila University of Medicine and Pharmacy, Bucharest, ROU; 10 Department of Biophysics and Biostatistics, Ovidius University of Constanta - Faculty of Dental Medicine, Constanta, ROU; 11 Department of Biostatistics, Ovidius Doctoral School of Medicine, Constanta, ROU; 12 Department of Pneumology, Ovidius University of Constanta - Faculty of Medicine, Constanta, ROU; 13 Department of Pneumology, Ovidius Doctoral School of Medicine, Constanta, ROU

**Keywords:** intermittent hypoxia-hyperoxia therapy, medical rehabilitation, metabolic effects, obesity, respiratory effects

## Abstract

Introduction

Obesity is a complex condition characterized by excessive accumulation of body fat, which can have multiple causes, including genetic factors, inadequate diet, lack of physical exercise, and socioeconomic factors. Obesity can cause significant respiratory changes, so obese patients present pulmonary complications more frequently than individuals with normal weight. Improving respiratory function is an important aspect of obesity management, as it can reduce the risk of pulmonary complications and improve patients' quality of life.

Material and method

We conducted a randomized controlled, single-center study that included 70 obese patients, aiming to evaluate the effectiveness of intermittent hypoxia-hyperoxia therapy (IHHT) on metabolic and respiratory effects. Patients were randomly allocated into two equivalent groups: an intervention group, consisting of 35 patients who received IHHT, and a control group, consisting of 35 patients who did not receive this therapy.

Results

Patients in the intervention group showed a significant increase in exercise tolerance (p < 0.001), improvement in renal function parameters (p = 0.047 for uric acid; p = 0.006 for creatinine), and liver function (p = 0.001 AST; p = 0.030 ALT), compared to the control group. An improvement in the Tiffeneau index was also observed in the intervention group (p < 0.001), indicating an improvement in respiratory function and lung capacity.

Conclusions

The approach to obesity requires a holistic perspective that takes into account the physical, psychological, and social aspects of this condition. IHHT represents an integrative therapeutic approach that addresses both the metabolic and respiratory aspects of obesity and metabolic syndrome, offering promising prospects for improving patients' health and quality of life. The study results suggest that IHHT may be effective in improving physical performance, renal and hepatic function, as well as respiratory function, with the potential to provide significant benefits in the management and treatment of obese and/or metabolic syndrome patients.

## Introduction

Obesity represents the excessive or abnormal accumulation of adipose tissue in the body, which affects health through its association with the risk of developing diabetes mellitus, cardiovascular and pulmonary diseases, and hypertension. According to the World Health Organization (WHO), over 650 million adults were classified as obese globally in 2021, representing 13% of the world’s population, with the prevalence continuing to rise across all regions [1]. It is the second leading preventable cause of death after smoking. Even a modest weight loss of 5% to 10% can significantly enhance health, improve quality of life, and reduce the economic burden on both individuals and society [1]. It is defined by a body mass index (BMI) of 30 kg/m² or higher, calculated by dividing a person's weight by the square of their height [2].

Obesity is one of the most challenging issues in medical practice, with its prevalence increasing despite the efforts of both patients and doctors. Treatment requires a multimodal approach that varies depending on the stage of the disease and its goals (e.g., weight loss and weight maintenance). The primary therapy for obesity should involve a non-surgical approach that includes appropriate nutrition, physical exercise, and behavior change [3,4]. Obesity is a significant risk factor for pulmonary pathologies such as asthma, obstructive sleep apnea (OSA), obesity hypoventilation syndrome, and pulmonary hypertension. Obesity increases susceptibility to respiratory infections and hospitalization rates are higher in obese patients with respiratory diseases compared to healthy weight subjects [5,6].

The impact of obesity on pulmonary function is multifactorial, linked to both mechanical and inflammatory aspects of obesity. Obesity causes substantial changes in lung and chest wall mechanics, and these mechanical changes lead to asthma and asthma-like symptoms such as dyspnea, wheezing, and airway hyperresponsiveness. These mechanical effects are not easily quantified with conventional pulmonary function testing and body mass index measurement. Changes in mediators produced by adipose tissue also contribute to changes in lung function, although this aspect is still poorly understood to date [7-9].

Intermittent hypoxia (IH) involves alternating between repeated episodes of hypoxia and normoxia [10]. IH-hyperoxia is a therapy that uses hyperoxic intervals instead of normoxic ones between hypoxic breathing sessions. The patient receives a gas mixture containing 30%-40% O_2_ through a mask [11]. Studies conducted over the years with IH therapy have demonstrated increased exercise tolerance in patients with cardiovascular, bronchopulmonary, and metabolic syndromes, improved cardiometabolic status in geriatric patients, and increased cognitive potential in Alzheimer's disease [12].

Obesity requires prolonged pharmacological treatment, which may not be convenient or accessible for many patients. Lifestyle modification, nutraceuticals, patient education, and other promising new therapies can limit side effects and improve patient compliance. In this context, intermittent passive exposure to hypoxia during rest has been identified as a promising strategy. This method may reduce cardiometabolic risk factors and be beneficial in treating patients with difficulties in physical activities and those who want to adopt a healthy lifestyle [13-15].

This study aimed to evaluate the effectiveness of IH-hyperoxia therapy (IHHT) among obese patients, focusing on metabolic and respiratory effects. It aimed to determine if this method could benefit managing or alleviating the complications associated with obesity. Assessing the effectiveness of IHHT aims to understand the impact of this therapy on the metabolic and respiratory health of selected patients, providing important insights for managing and improving their health status.

## Materials and methods

Study design

We conducted a randomized, controlled, unicentric study involving 70 obese patients (BMI>30 kg/m^2^). Patients were randomly allocated into two equivalent groups: 35 patients in the intervention group who received IHHT, and 35 patients in the control group who did not receive this therapy.

The study started after receiving approval from the Balneal and Rehabilitation Sanatorium ethics committee of Techirghiol, no. 6403/28.04.2023. The study was conducted in accordance with the ethical standards established in the Helsinki Declaration - Ethical Principles for Medical Research Involving Human Subjects. All subjects initially read the consent form, which informed them about the study, its purpose, and what it entailed. Subsequently, all patients signed the patient`s informed consent form.

This clinical study on IHHT in obese patients has been officially registered on the ClinicalTrials.gov website under the identification number NCT06451601. The study focuses on evaluating the effectiveness and safety of this innovative therapy in obese patients [16].

Participants and randomization

The study was conducted at the Department of Adult Rehabilitation from Balneal and Rehabilitation Sanatorium of Techirghiol between April 1 and May 15, 2024, and involved 92 cardiorespiratory-stable obese patients aged between 40 and 75. According to the WHO, obesity is a BMI ≥ 30 kg/m^2^ [2].

The inclusion criteria were as follows: patients with a BMI >30 kg/m^2^, aged between 40 and 75, patients who signed the informed consent form, and patients who were compensated and receiving medical treatment for associated pathologies.

The exclusion criteria were as follows: BMI < 30 kg/m^2^, age under 40 or over 75 years, refusal to sign the informed consent form, patients presenting contraindications to IHHT, and patients presenting contraindications to complex medical rehabilitation treatment.

**Figure 1:**
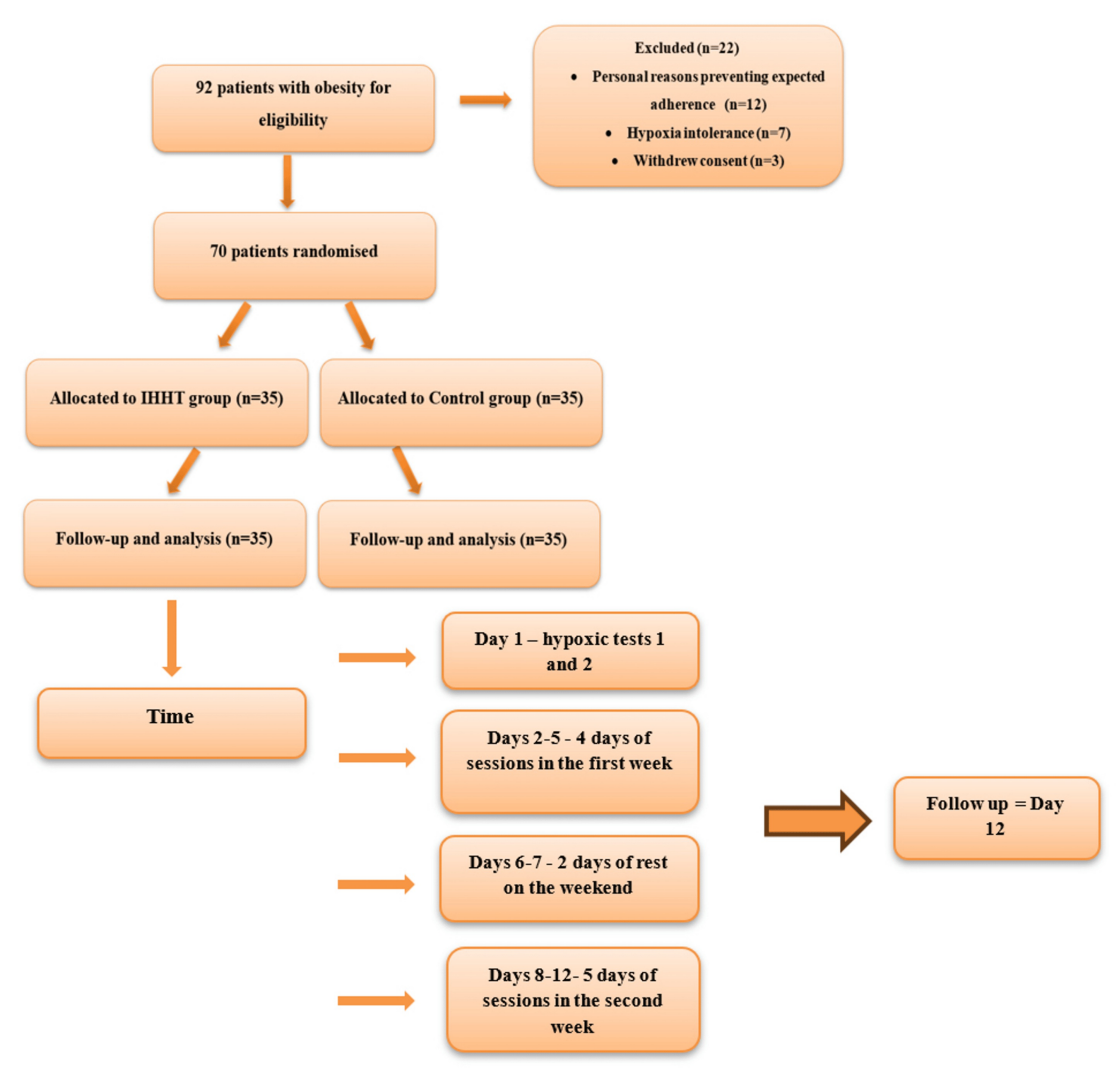
Design study flowchart

After excluding 22 patients, 70 patients were randomly allocated (by drawing lots) either to the IHHT group (35 patients) or to the control group (those who did not receive IHHT or sham) (35 patients). The inclusion process, randomization, stratification, hypoxia-hyperoxia program, and outcome analysis are presented in Figure 1. Patients were asked to maintain their daily food intake, physical activity, prescribed medications, and usual lifestyle throughout the study period.

IH-hyperoxia therapy

**Figure 2:**
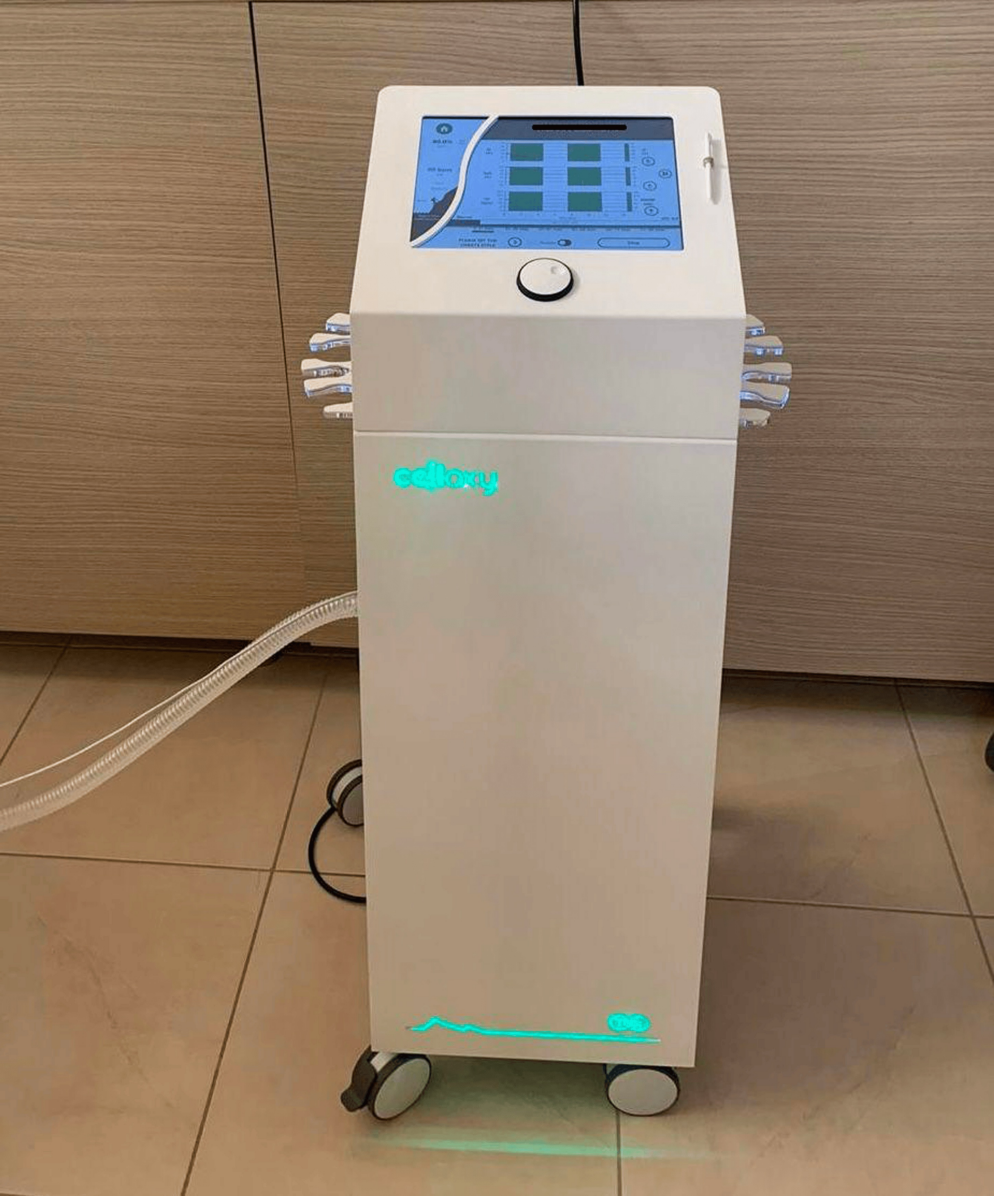
CellOxy device used for intermittent hypoxia-hyperoxia therapy

Simultaneously with the IHHT, the patients benefited from complex balneo-physical-kinetic treatment which included hydrokinetotherapy and hydrothermotherapy using specific natural environmental factors: sapropelic mud and salt water from Lake Techirghiol, electrotherapy, massage therapy and kinetotherapy. Thirty-five patients in the IHHT group underwent IHHT using the CellOxy Device (CellOxy, Germany) (Figure 2) [17].

Patients received 9%-16% O_2_ concentrations during the hypoxic phases, while approximately 35% O_2_ was administered during the hyperoxic phases. During both testing and therapy sessions, patients remained in a comfortable position, lying on a bed. Initially, patients performed hypoxic tests 1 and 2, to determine the optimal oxygen level and to be included in a typology (type I, II, or III). The device automatically calculated and planned personalized IHHT sessions for each patient based on the obtained data.

Hypoxic test 1's approximate duration is two to 10 minutes. This test classifies patients into three typologies (type I, type II, type III). Type I represents the typology with low resistance, which means these patients need additional assistance, while type III shows high resistance. This test helps identify the minimum duration of the hypoxic interval (phase 1) and the minimum duration of the normoxia/hyperoxia interval (phase 2).

The approximate duration of hypoxic test 2 is three to 10 minutes. This test helps to establish the optimal O2 level (between 9% and 16%), being an automatic test. Hypoxic test 2 is performed according to the target oxygen saturation (for the period of hypoxia). About 85% (elderly) is initially recommended for type I and II patients. For type III, those with high resistance - 80% (healthy adult) [17].

Starting from the following day, patients in the IHHT group were subjected to intermittent hypoxia-hyperoxia as follows: hypoxia with 9%-16% O_2_ for five to seven minutes, followed by exposure to hyperoxia with ~35% O_2_ for two to five minutes. SpO_2_ and heart rate were constantly monitored during the sessions using the device's built-in pulse oximeter.

In total, patients in the IHHT group performed nine sessions of hypoxic-hyperoxic therapy: testing on the first day (hypoxic tests 1 and 2), followed by four days of sessions in the first week, two days of rest on the weekend and then another five days of sessions in the second week.

During the IHHT, no adverse reactions were recorded among the studied patients. The control group completed only the comprehensive balneo-physical-kinetic treatment without IHHT, neither real nor simulated.

Procedures and assessments

The initial assessment of patients consisted of a comprehensive medical examination, which identified their comorbidities, family history, verification of medication for associated pathologies, and behavioral factors (smoking, alcohol consumption, psychosocial stress).

All patients underwent the following measurements: resting blood pressure (BP), heart rate (HR), blood oxygen saturation (SpO_2_), anthropometric data: height (cm), body weight (kg), waist circumference (cm), and hip circumference (cm), BMI calculation, blood sampling (urea, uric acid, creatinine, glucose, total cholesterol, AST, ALT). Regardless of the presence or absence of changes in the analysis results, patients were included in the study.

We consider it important to investigate the entire spectrum of the studied patients, including those with evident changes in their analyses and those with normal results, to obtain a comprehensive and representative image of the researched phenomenon. The patients also performed a six-minute walk test (6WT) and a spirometry. After completing 12 days of hospitalization at the Balneal and Rehabilitation Sanatorium of Techirghiol, the final assessment identified the same parameters as followed initially.

Resting heart rate and blood oxygen saturation values were measured using the Beurer PO 30 pulse oximeter (Beurer GMBH, Germany). Blood pressure was measured using a Moretti DM345 mechanical tensiometer. Body weight was measured using the Tanita RD-953 scale. Waist circumference was measured at the midpoint between the lower edge of the last palpable rib and the top of the iliac crest. Hip circumference was measured at the widest part of the hip.

Venous blood samples were drawn from the median brachial vein of the forearm and collected into vacuum tubes for analysis. The analyses were performed at the analysis laboratory within the Balneal and Rehabilitation Sanatorium of Techirghiol.

Statistical methods

The statistical analysis was performed using IBM SPSS statistics software version 25 (IBM Corp., Armonk, NY). Data are presented as mean ± standard deviation (SD) for continuous variables in case of symmetric distributions, median, and IQR (Interquartile range IQR = P75-P25) for numerical discrete variables or continuous variables in case of skewed distributions, or as frequencies and percentages for categorical variables. The normality of the continuous data was estimated with Shapiro-Wilk tests of normality. For hypotheses testing, independent samples Mann-Whitney U test, related samples Wilcoxon signed rank test, chi-square test of association, and z-test for the comparison of two proportions were used depending on the type of analyzed variables. The significance level (α) was set at 0.05. If the test statistic for any conducted test fell within the critical region and the p-value was less than or equal to the significance level, we rejected the null hypothesis in favor of the alternative hypothesis.

## Results

The descriptive statistics, comparisons, and differences provide crucial information for understanding and interpreting the study results. These data help identify baseline differences between the studied groups and relevant prognostic factors. Interpreting the clinical significance of these results is essential for their application in medical practice and for formulating effective, evidence-based protocols.

Table 1 presents the demographic characteristics of the interventional and control groups, showing no significant intergroup differences across the assessed variables. However, notable intragroup differences were observed: the control group showed a significant gender imbalance with more women than men (z = 2.630, p = 0.009); the intervention group had significantly more urban patients compared to rural patients (z = 3.586, p < 0.001); and both groups exhibited a higher proportion of non-smokers over smokers within themselves (intervention group: z = 5.758, p < 0.001; control group: z = 5.507, p < 0.001).

**Table 1:** Demographic data of the interventional and cntrol groups z-value: the number of standard deviations a data point is from the mean p-value: statistical measure that helps determine whether the observed results are statistically significant.

		Interventional (35)		Control (35)		Total		z-value	P-value
Sex	Female	20	57.14%	23	65.71%	43	61.43%	-0.737	0.461
	Male	15	42.86%	12	34.29%	27	38.57%	0.737	0.461
Environment	Urban	25	71.43%	18	51.43%	43	61.43%	1.719	0.086
	Rural	10	28.57%	17	48.57%	27	38.57%	-1.719	0.086
Occupation	Retired	19	54.29%	17	48.57%	36	51.43%	0.478	0.632
	Employed	16	45.71%	18	51.43%	34	48.57%	-0.478	0.632
Psychosocial stress	Yes	19	54.29%	18	51.43%	37	52.86%	-0.239	0.811
	No	16	45.71%	17	48.57%	33	47.14%	0.239	0.811
Lifestyle	Sedentary	7	20.00%	5	14.29%	12	17.14%	0.634	0.526
	Active	28	80.00%	30	85.71%	58	82.86%	-0.634	0.526
Smoking	Yes	4	11.43%	5	14.29%	9	12.86%	-0.357	1
	No	28	80.00%	28	80.00%	56	80.00%	0	1
	Former smoker	3	8.57%	2	5.71%	5	7.14%	0.464	0.643
Alcohol	Yes	1	2.86%	5	14.29%	6	8.57%	-0.718	0.473
	No	15	42.86%	18	51.43%	33	47.14%	-0.718	0.473
	Occasional	19	54.29%	12	34.29%	31	44.29%	1.684	0.092
Obesity	Grade I	25	71.43%	28	80.00%	53	75.71%	-0.836	0.403
	Grade II	7	20.00%	7	20.00%	14	20.00%	0	1
	Grade III	3	8.57%	0	0.00%	3	4.29%	1.770	0.077

Table 2 reveals a notable pattern in how the intervention impacted various health parameters. Participants who received the intervention showed significant improvements in their 6WT distances. They also exhibited beneficial changes in biochemical markers, including reductions in uric acid and creatinine levels, as well as improved liver enzymes (AST and ALT), Additionally, the Tiffeneau index improved significantly in the intervention group, suggesting respiratory benefits. In contrast, the intervention did not appear to influence glucose or total cholesterol levels, as no significant differences were observed between the groups in these areas.

**Table 2:** Descriptive statistics for key biomarkers and functional tests at admission and discharge in interventional and control groups 6WT: Six-Minute Walk Test: functional test that measures the distance, in meters, walked in six minutes to assess aerobic capacity and endurance Uric Acid (mg/dL): marker of kidney function Creatinine (mg/dL): marker of kidney health and function Glucose (mg/dL): blood sugar levels Total Cholesterol (mg/dL): measure of cholesterol in the blood AST (U/L): Aspartate Aminotransferase, a liver enzyme indicating liver function ALT (U/L): Alanine Aminotransferase, a liver enzyme indicating liver function Tiffeneau Index: spirometry measure for lung function (FEV1/FVC ratio) HTi: Hypoxia Training Index, measures dosage of hypoxia exposure during therapy N: Number of participants in each group SD: Standard deviation, a measure of the variation in the data Min/Max: The minimum and maximum values in the group P25, Median, P75: the 25th percentile, median, and 75th percentile of the data, representing the distribution of the values

	Group		Mean	SD	Min	Max	Percentiles		
		N					P25	Median	P75
6WT distance at admission (m)	Interventional	35	394.09	48.08	314.00	486.00	354.00	392.00	439.00
	Control	35	412.26	68.36	280.00	598.00	369.00	400.00	453.00
6WT distance at discharge (m)	Interventional	35	426.69	57.53	317.00	534.00	373.00	426.00	470.00
	Control	35	407.14	65.78	320.00	650.00	358.00	400.00	442.00
Uric acid at admission (mg/dL)	Interventional	35	5.80	1.58	3.44	9.02	4.61	5.49	7.21
	Control	35	5.61	1.35	3.67	4.34	4.48	5.48	6.62
Uric acid at discharge (mg/dL)	Interventional	35	6.48	1.43	3.06	8.27	8.26	5.22	6.81
	Control	35	5.58	1.32	3.27	8.82	4.84	5.25	6.01
Creatinine at admission (mg/dL)	Interventional	35	1.18	0.16	0.76	1.56	1.09	1.16	1.26
	Control	35	1.13	0.18	0.79	1.68	1.02	1.14	1.21
Creatinine at discharge (mg/dL)	Interventional	35	1.08	0.14	0.80	1.32	.99	1.04	1.23
	Control	35	1.11	0.18	0.69	1.57	1.01	1.08	1.27
Glucose at admission (mg/dL)	Interventional	35	120.60	27.73	83.00	180.00	101.00	112.00	144.00
	Control	35	119.34	26.03	88.00	183.00	102.00	113.00	132.00
Glucose at discharge (mg/dL)	Interventional	35	116.91	23.16	81.00	183.00	102.00	112.00	131.00
	Control	35	114.37	20.38	83.00	162.00	100.00	109.00	130.00
Total cholesterol at admission (mg/dL)	Interventional	35	202.54	52.00	113.00	326.00	172.00	190.00	247.00
	Control	35	216.29	46.08	125.00	315.00	180.00	216.00	252.00
Total cholesterol at discharge (mg/dL)	Interventional	35	189.43	47.50	97.00	295.00	153.00	188.00	216.00
	Control	35	209.49	44.82	130.00	317.00	170.00	207.00	238.00
AST at admission (U/L)	Interventional	35	29.46	12.54	16.00	73.00	21.00	25.00	32.00
	Control	35	24.71	7.63	10.00	44.00	19.00	24.00	28.00
AST at discharge (U/L)	Interventional	35	26.71	12.48	10.00	66.00	20.00	23.00	28.00
	Control	35	23.94	5.97	12.00	39.00	19.00	23.00	28.00
ALT at admission (U/L)	Interventional	35	32.57	18.53	12.00	96.00	20.00	27.00	41.00
	Control	35	26.60	11.02	11.00	63.00	18.00	25.00	33.00
ALT at discharge (U/L)	Interventional	35	32.40	19.59	8.00	92.00	19.00	26.00	38.00
	Control	35	26.09	9.45	11.00	48.00	20.00	24.00	31.00
Tiffeneau index at admission	Interventional	35	88.26	08.07	66.93	98.28	82.99	90.42	94.62
	Control	35	92.48	7.79	71.33	100.00	89.26	94.34	98.85
Tiffeneau index at discharge	Interventional	35	92.92	7.39	71.08	100.00	88.29	95.57	99.01
	Control	35	91.66	7.71	70.86	100.00	88.02	92.85	98.82
HTi at admission	Interventional	35	68.00	28.20	8.00	144.00	49.00	67.00	83.00
HTi at discharge	Interventional	35	94.29	34.26	20.00	166.00	75.00	88.00	116.00

Table 3 provides an overview of continuous variables evolution in the interventional and control groups, from admission to discharge. Aside from key indicators such as the 6WT distance, diverse biochemical parameters, and Tiffeneau, measures of the Hypoxia Training Index (HTi) are presented. Each variable is depicted with means, standard deviations, minimum, maximum, and percentile values, allowing for an in-depth comparison of the groups' progress over time.

**Table 3:** Frequency distribution of improvements in functional and biochemical parameters in interventional and control groups 6WT – Six-Minute Walk Test: functional test that measures the distance, in meters, walked in six minutes to assess aerobic capacity and endurance IHHT – Intermittent Hypoxia-Hyperoxia Therapy:  therapeutic intervention that alternates between hypoxia (low oxygen levels) and hyperoxia (high oxygen levels) to stimulate physiological adaptation AST – Aspartate Aminotransferase: liver enzyme used to assess liver function ALT – Alanine Aminotransferase: liver enzyme used to assess liver function Tiffeneau Index –  ratio used in spirometry to measure lung function, also known as the FEV1/FVC ratio HTi – Hypoxia Training Index:  measure of the dosage of hypoxia exposure during therapy sessions p – value:  statistical measure that helps determine whether the observed results are statistically significant p < 0.05 (*): statistically significant p < 0.01 (**): highly statistically significant Chi-Square value – statistic used to determine whether there is a significant association between two categorical variables by comparing observed and expected frequencies

		Interventional (35)		Control (35)		Total		Chi-square	P-value
Improvement of 6WT distance	Yes	33	94.29%	11	31.43%	44	62.86%	29.615	<0.001**
	No	2	5.71%	24	68.57%	26	37.14%		
Improvement of uric acid	Yes	24	68.57%	16	45.71%	40	57.14%	3.925	0.047*
	No	11	31.43%	19	54.29%	30	42.86%		
Improvement of creatinine	Yes	28	80.00%	17	48.57%	45	64.29%	7.529	0.006*
	No	7	20.00%	18	51.43%	25	35.71%		
Improvement of AST	Yes	27	77.14%	13	37.14%	40	57.14%	11.433	0.001**
	No	8	22.86%	22	62.86%	30	42.86%		
Improvement of ALT	Yes	20	57.14%	11	31.43%	31	44.29%	Apr.69	0.030*
	No	15	42.86%	24	68.57%	39	55.71%		
Improvement of Tiffeneau Index	Yes	33	94.29%	10	28.57%	43	61.43%	31.895	<0.001**
	No	2	5.71%	25	71.43%	27	38.57%		
Improvement of glucose	Yes	25	71.43%	21	60.00%	46	65.71%	1.014	0.314
	No	10	28.57%	14	40.00%	24	34.29%		
Improvement of total cholesterol	Yes	24	68.57%	18	51.43%	42	60.00%	2.143	0.143
	No	11	31.43%	17	48.57%	28	40.00%		

**Figure 3:**
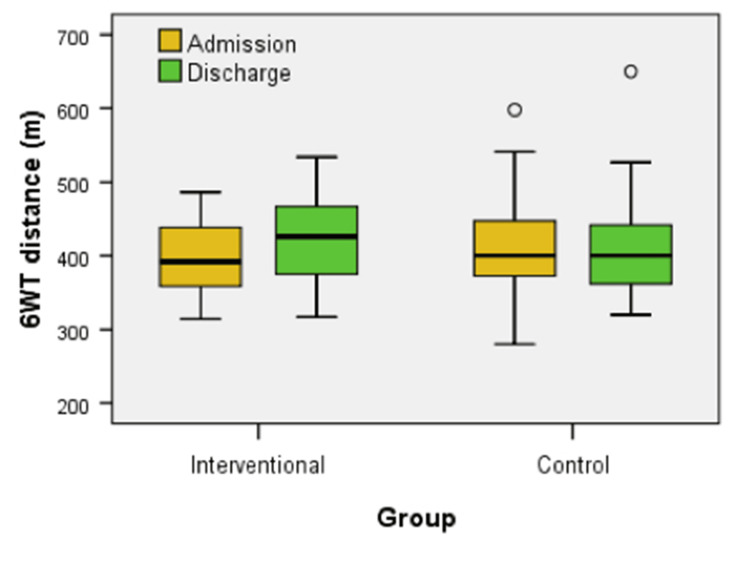
Box plot comparison of six-minute walk test (6WT) distance at admission and discharge between interventional and control groups The 6WT is a functional test that measures the distance, in meters, walked in six minutes to assess aerobic capacity and endurance.

Figure 3 illustrates the 6WT distances at admission and discharge for both the interventional group, which received IHHT, and the control group (U = 526.00, z = -1.016, p = 0.310). While no significant differences were observed between the groups at either time point (U = 526.00, z = -1.016, p = 0.310 - admission, U = 463.00, z = -1.756, p = 0.079 - discharge), a significant improvement occurred within the interventional group throughout the treatment (z = -4.636, p < 0.001).

**Figure 4:**
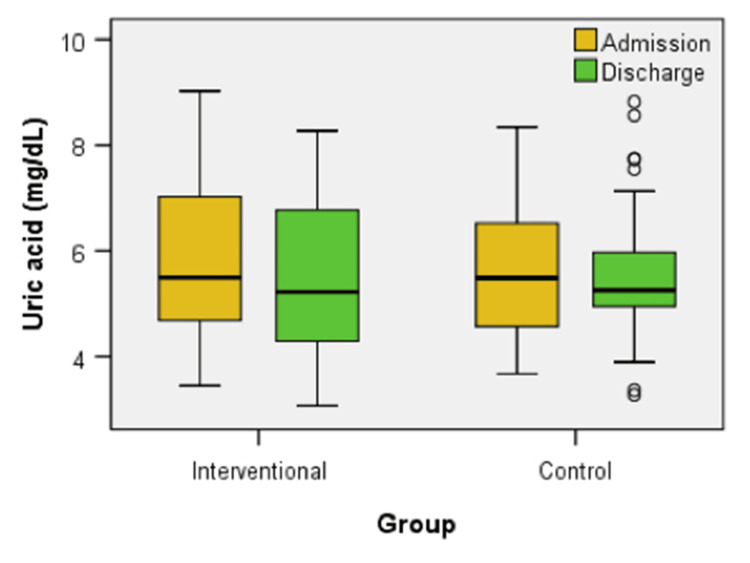
Box plot comparison of uric acid levels at admission and discharge between interventional and control groups

Figure 4 displays the uric acid levels at admission and discharge for both the interventional and control groups. There were no significant differences between the groups at either time point (U = 580.00, z = -0.382, p = 0.703 - admission, U = 585.50, z = -0.317, p = 0.751 - discharge). However, within the interventional group, uric acid levels decreased significantly from admission to discharge (z = -2.613, p = 0.009), indicating a positive response to the intervention (z = -0.147, p = 0.883). In contrast, the control group showed no significant change in uric acid levels over the same period (z = -0.147, p = 0.883).

**Figure 5:**
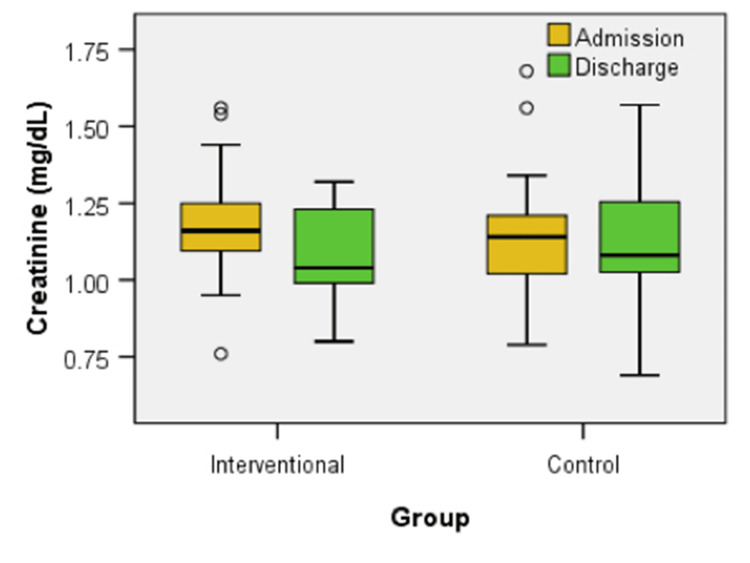
Box plot comparison of creatinine levels at admission and discharge between interventional and control groups

Figure 5 illustrates the creatinine levels at admission and discharge for both the interventional and control groups. No significant differences were observed between the groups at either time point (U = 484.50, z = -1.504, p = 0.133). However, within the interventional group, creatinine levels decreased significantly from admission to discharge (z = -4.303, p < 0.001). In contrast, the control group showed no significant change in creatinine levels over the same period.

**Figure 6:**
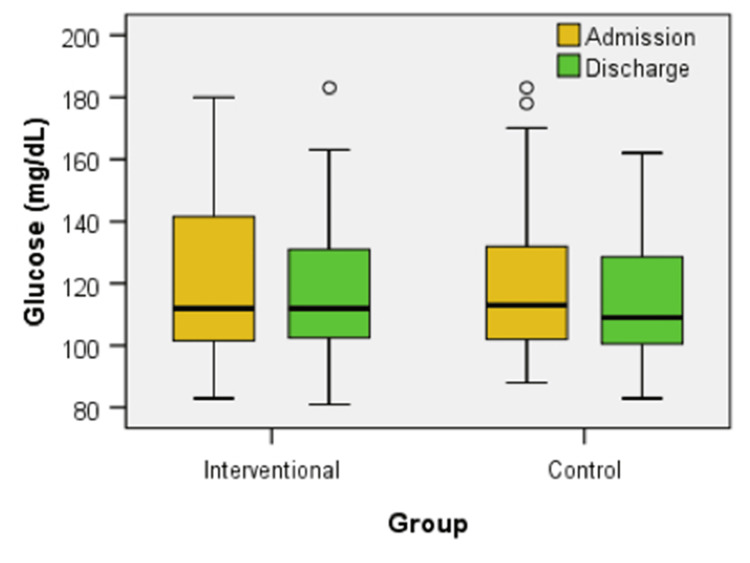
Box plot comparison of glucose levels at admission and discharge between interventional and control groups

Figure 6 shows the glucose levels at admission and discharge for both groups. No difference was observed between the groups at either time point (U = 611.00, z = -0.018, p = 0.986 - admission, U = 574.50, z = -0.447, p = 0.655 - discharge). Within the interventional group, there was a statistically significant decrease in glucose levels from admission to discharge (z = -2.163, p = 0.031). The control group did not show a significant change (z = -1.882, p = 0.060).

**Figure 7:**
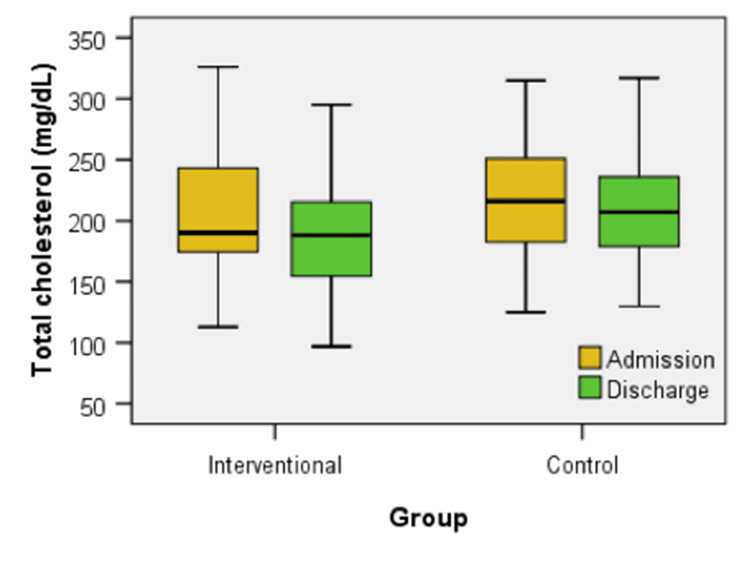
Box plot comparison of total cholesterol levels at admission and discharge between interventional and control groups

Figure 7 illustrates the box-plot distribution of total cholesterol levels at admission and discharge for both the interventional and control groups. The Mann-Whitney U test revealed no significant differences in total cholesterol levels between the groups at admission (U = 520.00, z = -1.087, p = 0.277) or discharge (U = 468.00, z = -1.697, p = 0.090). However, within the interventional group, a Wilcoxon signed-rank test showed a statistically significant decrease in total cholesterol levels from admission to discharge (z = -2.095, p = 0.036). In contrast, the control group did not exhibit a significant change over the same period (z = -1.049, p = 0.294).

**Figure 8:**
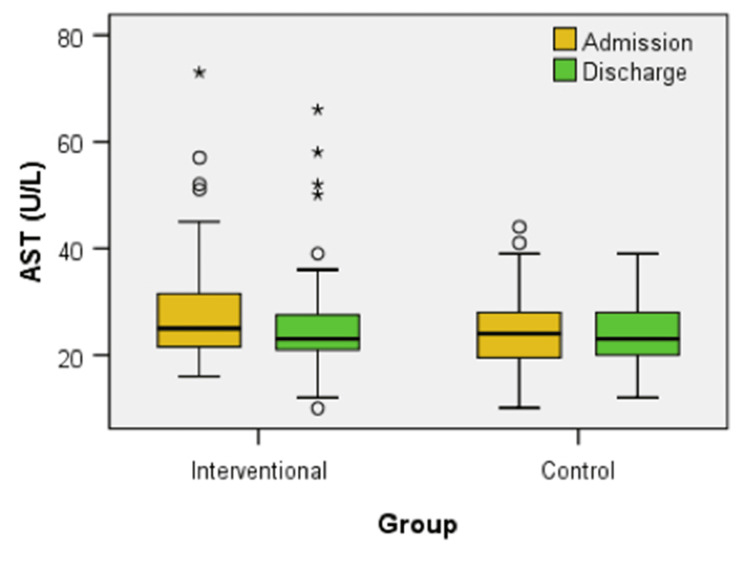
Box plot comparison of AST levels at admission and discharge between interventional and control groups

Figure 8 provides the box-plot distribution of AST levels at admission and discharge for both the interventional and control groups. The Mann-Whitney U test revealed no significant differences in AST levels between the groups at admission (U = 485.00, z = -1.500, p = 0.134) or discharge (U = 612.50, z < -0.001, p = 1.000). However, within the interventional group, a Wilcoxon signed-rank test showed a statistically significant decrease in AST levels from admission to discharge (z = -3.136, p = 0.002), indicating a positive effect of the treatment. In contrast, the control group did not exhibit a significant change over the same period (z = -0.485, p = 0.628).

**Figure 9:**
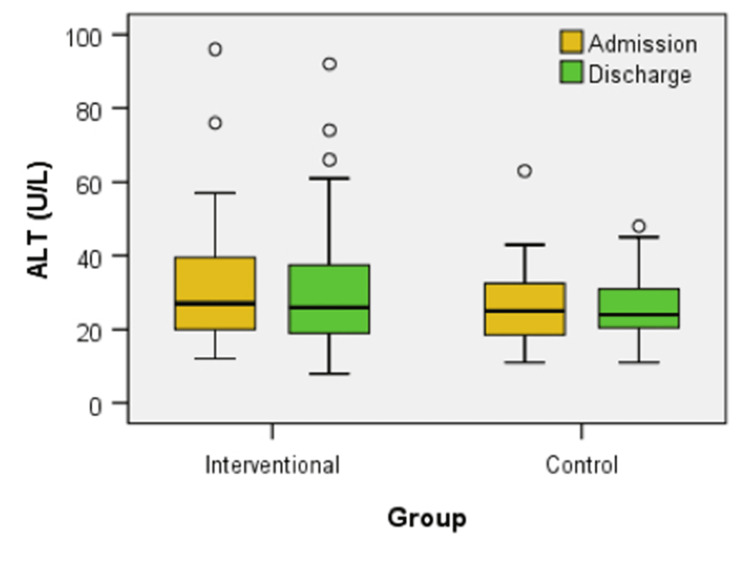
Box plot comparison of ALT levels at admission and discharge between interventional and control groups

Figure 9 displays the box-plot distribution of ALT levels at admission and discharge for both the interventional and control groups. No significant differences were found in ALT levels between the groups at admission (U = 519.00, z = -1.099, p = 0.272) or at discharge (U = 537.00, z = -0.888, p = 0.375). Additionally, the treatment did not elicit a statistically significant change in ALT levels within the interventional group (z = -0.855, p = 0.393) or the control group (z = -0.707, p = 0.479) from admission to discharge.

**Figure 10:**
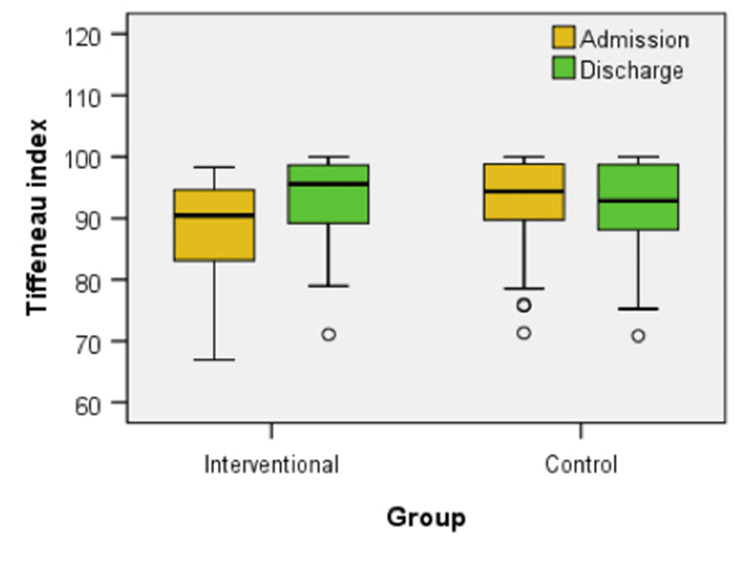
Box plot comparison of Tiffneau index at admission and discharge between interventional and control groups

Figure 10 illustrates the box-plot distribution of the Tiffeneau index at admission and discharge for both the interventional and control groups. No significant differences in the Tiffeneau index were found between the groups at admission (U = 467.00, z = -1.709, p = 0.082) or discharge (U = 548.00, z = -0.760, p = 0.448). The interventional group showed a statistically significant improvement in the Tiffeneau index from admission to discharge (z = -4.504, p < 0.001). The control group did not exhibit a significant change over the same period (z = -0.978, p = 0.328).

**Figure 11:**
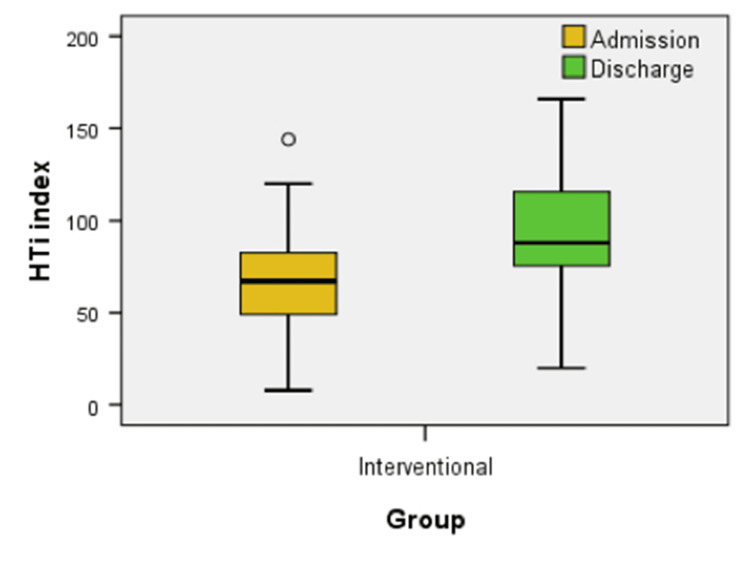
Box plot comparison of HTi at admission and discharge between interventional and control groups HTi - Hypoxia Training Index

Figure 11 displays the HTi values at admission and discharge for the interventional group. We noted a statistically significant increase in HTi values from admission to discharge (z = -3.456, p = 0.001).

## Discussion

Obesity is widely recognized for its significant association with cardiovascular diseases, but its impact on respiratory function has been less explored [1,2,5]. Given the rising prevalence of obesity globally, it is crucial to address the complex interplay between obesity and respiratory health. Studies have shown that obesity is an independent risk factor for a range of respiratory complications, including OSA, asthma, and obesity hypoventilation syndrome (OHS), highlighting the necessity of investigating therapeutic interventions that can improve pulmonary outcomes in this population [18-20].

Obesity plays a key role in the development of OSA and obesity hypoventilation syndrome [21]. Asthma is more common and often more difficult to treat in the obese population [22]. Also, the literature has discussed the effects of obesity on chronic obstructive pulmonary disease (COPD) and the paradoxical interaction between body mass index and COPD severity [23].

Recent data suggest that the prevalence of wheezing and bronchial hyperreactivity, two symptoms often associated with asthma, are increased in overweight and obese individuals [24]. Epidemiological studies have reported that obesity is a risk factor for asthma development [25]. Furthermore, many studies indicate that obesity is also associated with a higher risk of developing deep vein thrombosis, pulmonary embolism, pulmonary hypertension, and pneumonia [26-28]. Ultimately, weight loss has been proven effective in improving symptoms and severity of several respiratory diseases, including OSA and asthma [29,30]. Obese patients should be encouraged to lose weight to reduce their risk of developing respiratory diseases or to improve preexisting conditions [31].

The safety and efficacy of IHHT demonstrated in this study are supported by the literature through the successful use of IHHT in various pathologies [32-37]. Before starting the present study, patients were randomized into intervention and control groups, ensuring that factors influencing the results were evenly distributed between the two groups. Analysis of baseline characteristics of patients in both groups showed no significant differences regarding age, gender, environment, occupation, or other important variables. This is essential to minimize the possibility of confounding and accurately interpret the study results. By having patients with similar characteristics in both groups, we can be more confident that any differences observed in outcomes are truly caused by the treatment used and not by other confounding variables. It is essential to highlight the baseline uniformity of patient characteristics in both groups, as this strengthens the validity and credibility of the study results. Thus, ensuring uniformity of baseline characteristics between groups is essential for the study's validity and for making clinical decisions based on these results.

It is essential to consider the influence of various variables on study results. These variables can significantly alter our conclusions and interpretations from the collected data. Gender can have a considerable impact on study results. Different medical conditions, treatment responses, and health behaviors can vary by gender. Similarly, age is a critical variable in many studies because different life stages are associated with significant biological and psychological changes. Children, young adults, middle-aged individuals, and seniors may have distinct characteristics influencing their health status, behaviors, and perceptions. Geographic context and living environment can affect access to resources, quality of life, and exposure to risk factors. For example, rural residents may have more limited access to specialized medical services than urban residents, which can influence health-related outcomes and treatments. Also, the differences observed in patients who consume alcohol, smoke, and have an unhealthy lifestyle are particularly relevant because these behaviors can significantly influence health and implicitly, research outcomes. By analyzing these factors, researchers can offer more precise and personalized recommendations, thus contributing to improving medical practices.

We evaluated the age distribution of the patients in the two participating groups. The results showed no statistically significant differences regarding age between the two groups. Although our patients have varied ages, the age distribution is similar in both groups. The lack of significant differences in age between groups ensures that any effects observed in the study can be directly attributed to the treatment or interventions tested and not to variations in the age of the participants.

The study's results showed no statistically significant differences between the two groups regarding patient gender. The lack of significant gender differences ensures that any effects observed in the study are not influenced by this factor, demonstrating that the results can be applied to both male and female patients.

The two groups had no statistically significant differences regarding the patient's urban or rural environment. This demographic uniformity is crucial to ensure the validity and relevance of study results. It is essential to have representatives from both backgrounds to assess the effectiveness and safety of treatment in diverse life contexts and socio-economic conditions.

The two groups had no statistically significant differences regarding the patients' occupational status. The balance in terms of occupation (retired and employed) reflects the study population's diversity and contributes to our findings' clinical relevance to a wider population.

The similarity of the two groups regarding psychosocial stress suggests that this factor did not have a different impact on the evolution of the patients in the two groups. Considering that stress can affect patients' mental and physical health, it is essential to consider this variable in their assessment and management. Thus, therapeutic approaches should be designed considering these aspects and provide adequate support for stress management to improve the overall evolution of patients.

The sedentary and active people percentages in the interventional and control groups are comparable. This uniformity in the lifestyle distribution suggests that there are no significant differences between the groups regarding the patient's physical activity levels. This similarity can help eliminate potential biases in interpreting the results and contribute to the validity of comparing the study groups. 

Both groups show a similar distribution in the percentages of smokers, non-smokers, and former smokers, indicating an adequate balance between them. This similarity is essential for ensuring a valid and relevant comparison between the results obtained from each group.

The discrepancies in alcohol consumption percentages between the two groups are small, indicating a similarity in alcohol consumption behavior among the patients included in the study. Alcohol consumption can influence the results of a study in various ways, depending on the objectives and variables studied. In the case of major discrepancies in alcohol consumption between the study groups, it could be a confounding factor that affects the results and the validity of the conclusions. For example, excessive alcohol consumption can negatively influence treatment response or potentiate the side effects of certain medications. In our case, since the differences are small, the impact on the study results may be less significant, but it still needs to be considered and controlled in statistical analyses to avoid deviations in data interpretation. 

The uniformity in the distribution of nutritional status suggests that the obesity factor at admission did not differ significantly between the two study groups. If there are major discrepancies in BMI between the study groups, it could influence the results in different ways, depending on the objectives and variables studied. For example, if one group has a significantly higher average BMI than the other, this could influence outcomes related to treatment response or the disease progression associated with obesity. It is important to assess and control these discrepancies in statistical analyses to interpret the data accurately and draw relevant conclusions from the study.

There is a statistically significant relationship in the intervention group regarding the improvement in the distance covered during the 6WT, with a p-value < 0.001. This indicates strong evidence that IHHT significantly influences the distance traveled in six minutes, leading to increased exercise tolerance in patients within the intervention group. The odds of finding patients with an improvement in 6WT distance in the intervention group are 36.00 times higher than in the control group (OR = 36.00), a statistically significant result with a 95% CI of 7.299 to 177.553. Additionally, the proportion of patients presenting improvement in 6WT distance in the intervention group is 3.00 times higher than in the control group (RR = 3.00), another statistically significant result, with a 95% CI of 1.827 to 4.927.

There is a statistically significant relationship in the intervention group regarding the improvement of uric acid, with a p-value < 0.047. The chance of finding patients with improvement of uric acid in the interventional group is considered 2.591 times higher than the chance of finding patients with improvement of uric acid in the control group: OR = 2.591, a statistically significant value, because the confidence interval is 95% CI = (1.127, 6.872). The proportion of patients presenting improvement of uric acid in the interventional group is considered 1.5 times higher than the proportion of patients presenting improvement of uric acid in the control group: Rr = 1.5, a statistically significant value because the confidence interval is 95% CI = (1.097, 2.294).

In the intervention group, a statistically significant correlation was observed regarding the improvement of creatinine values, with a p-value < 0.006. The chance of finding patients with improvement of creatinine in the interventional group is considered 4.235 times higher than the chance of finding patients with improvement of creatinine in the control group: OR = 4.235, a statistically significant value, because the confidence interval is 95% CI = (1.466, 12.235). The proportion of patients presenting improvement of creatinine in the interventional group is considered 1.647 times higher than the proportion of patients presenting improvement of creatinine in the control group: Rr = 1.647, a statistically significant value because the confidence interval is 95% CI = (1.127, 2.406).

Compared to the control group, significant improvements in hepatic metabolism were also found in the intervention group (p = 0.001 for AST and p = 0.030 for ALT). The chance of finding patients with improvement of AST in the Interventional group is considered 5.712 times higher than the chance of finding patients with Improvement of AST in the control group: OR = 5.712, a statistically significant value, because the confidence interval is 95% CI = (2.008, 16.244). The proportion of patients presenting improvement of AST in the interventional group is considered 2.007 times higher than the proportion of patients presenting improvement of AST in the control group: Rr = 2.007, a statistically significant value because the confidence interval is 95% CI = (1.302, 3.314). The chance of finding patients with improvement of ALT in the interventional group is considered 2.909 times higher than the chance of finding patients with improvement of ALT in the control group: OR = 2.909, a statistically significant value, because the confidence interval is 95% CI = (1.093, 7.739). The proportion of patients presenting improvement of ALT in the interventional group is considered 1.818 times higher than the proportion of patients presenting improvement of ALT in the control group: Rr = 1.818, a statistically significant value because the confidence interval is 95% CI = (1.031, 3.206).

The Tiffeneau index also showed a significant improvement, with statistical significance (p < 0.001) in the interventional group. The chance of finding patients with improvement of the Tiffeneau index in the interventional group is considered 41.250 times higher than the chance of finding patients with improvement of the Tiffeneau index in the control group: OR = 41.250, a statistically significant value because the confidence interval is 95% CI = (8.289, 205.267). The proportion of patients presenting improvement of the Tiffeneau index in the interventional group is considered 3.300 times higher than the proportion of patients presenting improvement of the Tiffeneau index in the control group: Rr = 3.300, a statistically significant value because the confidence interval is 95% CI = (1.942, 5.607).

The results suggest that IHHT positively improved the HTi in a significant proportion of patients included in the study, from the interventional group. In 2009, Bassovich and Serebrovskaya proposed a new approach to objectively quantify hypoxia dose - the HTi. This is calculated by analyzing the SaO_2_ curve during a hypoxic test, providing a more objective measure of the hypoxic stress delivered during the session, compared to a simple reliance on FiO_2_. HTi provides an index of the dosage received by the individual at the end of the session [38].

The chance of finding patients with improvement of glucose in the interventional group does not differ significantly from the chance of finding patients with improvement of glucose in the control group: OR = 1.667, a value without statistical significance, because the 95% CI = (0.615, 4.519) contains the value of 1 (equal chances). The proportion of patients presenting improvement of glucose in the interventional group does not differ significantly from the proportion of patients presenting improvement of glucose in the control group: Rr = 1.190, a value without statistical significance because the 95% CI = (0.846, 1.676) contains the value of 1 (equal chances).

The chance of finding patients with improvement of total cholesterol in the interventional group does not differ significantly from the chance of finding patients with improvement of total cholesterol in the control group: OR = 2.061, a value without statistical significance, because the 95% CI = (0.778, 5.458) contains the value of 1 (equal chances). The proportion of patients presenting improvement of total cholesterol in the interventional group does not differ significantly from the proportion of patients presenting improvement of total cholesterol in the control group: Rr = 1.333, a value without statistical significance because the 95% CI = (0.901, 1.974) contains the value of 1 (equal chances).

A study examining the impact of IH-hyperoxia on cardiometabolic risk factors and trimethylamine-N-oxide (TMAO) levels in patients with metabolic syndrome observed that IH-hyperoxia during rest leads to a tendency to decrease blood TMAO levels. This was accompanied by a more significant reduction in cardiometabolic and hepatic indicators of metabolic syndrome than in the placebo group. Patients with initially elevated TMAO levels during IHHE showed a significant reduction in TMAO, but a lesser degree of reduction in total cholesterol, low-density lipoproteins, and hepatic steatosis than in the subgroup of patients with normal baseline TMAO levels [39]. IH-hyperoxia may be considered an effective and safe technique for improving cardiometabolic health, probably by normalizing the gut microbiome, gut wall barrier function, and hepatic metabolism.

Another randomized controlled clinical trial has demonstrated that IHHT can be safely used in patients with metabolic syndrome. The decrease in lipid metabolism and inflammatory markers in the IHHT group supports the effectiveness of this intervention in reducing systemic inflammation and improving lipid profile in patients with metabolic syndrome, with a great influence over other associated pathological conditions and increasing the quality of life [15,40-43].

Despite the promising results, the study has several limitations. The relatively small sample size limits the generalizability of the findings. Additionally, the therapy duration was only 12 days, which raises questions about the long-term sustainability of the observed improvements.

We want to highlight several future directions that could expand and deepen our understanding of IHHT and its role in managing obesity-related complications.

First, extending patient follow-up will be crucial to determine the long-term sustainability of IHHT’s benefits, particularly its effects on body weight, metabolic health, and overall physical function. Prolonged observation would provide valuable insight into how well the improvements in metabolic parameters, such as lipid profiles and liver function, are maintained over time, and whether the positive outcomes seen in exercise tolerance can persist or even further improve with longer interventions.

Second, a deeper understanding of the biological mechanisms through which IHHT influences metabolism is essential. Investigating how IHHT modulates cellular pathways involved in energy regulation, inflammation, and metabolic homeostasis can offer new perspectives. Specifically, evaluating how this therapy affects the expression of genes and proteins associated with obesity, insulin sensitivity, and fat metabolism could yield critical data that advances our ability to tailor treatments to individual patients. This mechanistic insight will not only add to the specialized literature but could also guide clinicians in optimizing treatment protocols for obesity-related conditions.

Another essential aspect for future research is conducting comparative studies that evaluate IHHT against other established interventions for obesity management, such as dietary modifications, physical exercise, behavioral therapies, and pharmacological treatments. By directly comparing IHHT with these approaches, we can determine its relative effectiveness, identify potential synergies, and clarify the optimal therapeutic combination or sequencing. Such research will be key to developing comprehensive, multimodal strategies for weight management and metabolic improvement.

We also want to point to the idea of an integrated and multidisciplinary approach to obesity-related complications. For instance, one of our previous studies highlighted the potential of telerehabilitation in managing post-stroke spasticity during the COVID-19 pandemic, ensuring continuity of care, and improving patient outcomes when in-person therapy was not feasible [44]. Such findings suggest that tele-rehabilitation when combined with therapies like IHHT, could offer a more comprehensive strategy for managing obesity-related respiratory and metabolic challenges. This integrated approach may ensure consistent therapeutic interventions, similar to how telerehabilitation supported patients during the pandemic, and could improve overall patient care, especially in settings where access to in-person therapy is limited.

Lastly, these future directions hold the potential to facilitate the development of more effective, personalized therapeutic strategies. By better understanding IHHT’s role in the broader context of obesity treatment, we can refine our approach to rehabilitation, ensuring that interventions are tailored to each patient’s unique metabolic and physical needs. This will enhance not only the clinical outcomes but also the quality of life of patients struggling with obesity and its associated health risks.

## Conclusions

IHHT has been successfully used and has brought benefits to the studied patients, being a safe and well-tolerated therapy. Research in the literature suggests that this therapy could have more benefits for this category of patients.

By emphasizing the link between obesity and pulmonary health, scientific literature can contribute to the development of more efficient and personalized management strategies. Understanding the impact of obesity on the respiratory system could lead to appropriate interventions for preventing and treating respiratory conditions associated with obesity, thus improving the patient's quality of life and reducing the costs associated with medical care. These findings underscore the therapeutic potential of IHHT in managing metabolic syndrome and support the need for further research in this area to clarify the mechanisms involved, develop more effective therapeutic strategies, and identify optimal ways of implementing therapy within obesity management. Additionally, it is essential for this therapy to be integrated into a larger treatment plan and to be monitored by medical professionals specialized in obesity management.

## References

[1] Panuganti KK, Nguyen M, Kshirsagar RK (2023). Obesity. https://www.ncbi.nlm.nih.gov/books/NBK459357/.

[2] Lin X, Li H (2021). Obesity: epidemiology, pathophysiology, and therapeutics. Front Endocrinol (Lausanne).

[3] Thompson WG, Cook DA, Clark MM, Bardia A, Levine JA (2007). Treatment of obesity. Mayo Clin Proc.

[4] Bischoff SC, Schweinlin A (2020). Obesity therapy. Clin Nutr ESPEN.

[5] Dixon AE, Peters U (2018). The effect of obesity on lung function. Expert Rev Respir Med.

[6] Collins LC, Hoberty PD, Walker JF, Fletcher EC, Peiris AN (1995). The effect of body fat distribution on pulmonary function tests. Chest.

[7] Hegewald MJ (2021). Impact of obesity on pulmonary function: current understanding and knowledge gaps. Curr Opin Pulm Med.

[8] Beuther DA, Sutherland ER (2007). Overweight, obesity, and incident asthma: a meta-analysis of prospective epidemiologic studies. Am J Respir Crit Care Med.

[9] Ford ES, Cunningham TJ, Mercado CI (2014). Lung function and metabolic syndrome: findings of National Health and Nutrition Examination Survey 2007-2010. J Diabetes.

[10] Burtscher M, Gatterer H, Szubski C, Pierantozzi E, Faulhaber M (2010). Effects of interval hypoxia on exercise tolerance: special focus on patients with CAD or COPD. Sleep Breath.

[11] Glazachev OS (2013). Optimization of clinical application of interval hypoxic training. Biomed Eng.

[12] Uzun AB, Iliescu MG, Stanciu LE (2023). Effectiveness of intermittent hypoxia-hyperoxia therapy in different pathologies with possible metabolic implications. Metabolites.

[13] Glazachev OS, Zvenigorodskaia LA, Dudnik EN, Iartseva LA, Mishchenkova TV, Platonenko AV, Spirina GK (2010). Interval hypoxic-hyperoxic training in the treatment of the metabolic syndrome. Eksp Klin Gastroenterol.

[14] Costalat G, Lemaitre F, Tobin B, Renshaw G (2018). Intermittent hypoxia revisited: a promising non-pharmaceutical strategy to reduce cardio-metabolic risk factors?. Sleep Breath.

[15] Afina AB, Oleg SG, Alexander AB (2021). The effects of intermittent hypoxic-hyperoxic exposures on lipid profile and inflammation in patients with metabolic syndrome. Front Cardiovasc Med.

[16] (2024). Intermittent hypoxia-hyperoxia therapy in obese patients (IHHTOP). https://clinicaltrials.gov/study/NCT06451601?cond=NCT06451601&rank=1.

[17] (2024). About celloxy. https://www.cell-oxy.com/thedevice.

[18] Cortes-Telles A, Ortiz-Farias DL, Pou-Aguilar YN, Almeida-de-la-Cruz L, Perez-Padilla JR (2021). Clinical impact of obesity on respiratory diseases: a real-life study. Lung India.

[19] Mafort TT, Rufino R, Costa CH, Lopes AJ (2016). Obesity: systemic and pulmonary complications, biochemical abnormalities, and impairment of lung function. Multidiscip Respir Med.

[20] Shah NM, Kaltsakas G (2023). Respiratory complications of obesity: from early changes to respiratory failure. Breathe (Sheff).

[21] Liu C, Chen MS, Yu H (2017). The relationship between obstructive sleep apnea and obesity hypoventilation syndrome: a systematic review and meta-analysis. Oncotarget.

[22] Peters U, Dixon AE, Forno E (2018). Obesity and asthma. J Allergy Clin Immunol.

[23] Zammit C, Liddicoat H, Moonsie I, Makker H (2010). Obesity and respiratory diseases. Int J Gen Med.

[24] Masa JF, Pépin JL, Borel JC, Mokhlesi B, Murphy PB, Sánchez-Quiroga MÁ (2019). Obesity hypoventilation syndrome. Eur Respir Rev.

[25] Marko M, Pawliczak R (2018). Obesity and asthma: risk, control and treatment. Postepy Dermatol Alergol.

[26] Stein PD, Beemath A, Olson RE (2005). Obesity as a risk factor in venous thromboembolism. Am J Med.

[27] Kushnir M, Billett H (2024). Association of obesity and thromboembolic disease. Obesity and Lung Disease.

[28] Badlam JB (2024). Obesity, metabolic syndrome, and pulmonary hypertension. Obesity and Lung Disease.

[29] Juel CT, Ali Z, Nilas L, Ulrik CS (2012). Asthma and obesity: does weight loss improve asthma control? a systematic review. J Asthma Allergy.

[30] Tuomilehto HP, Seppä JM, Partinen MM (2009). Lifestyle intervention with weight reduction: first-line treatment in mild obstructive sleep apnea. Am J Respir Crit Care Med.

[31] Murugan AT, Sharma G (2008). Obesity and respiratory diseases. Chron Respir Dis.

[32] Serebrovska ZO, Serebrovska TV, Kholin VA (2019). Intermittent hypoxia-hyperoxia training improves cognitive function and decreases circulating biomarkers of Alzheimer's disease in patients with mild cognitive impairment: a pilot study. Int J Mol Sci.

[33] Mallet R, Burtcher J, Manukhina E (2020). Hypoxic-hyperoxic conditioning and dementia. Diagn Manage Dement.

[34] Behrendt T, Bielitzki R, Behrens M, Herold F, Schega L (2022). Effects of intermittent hypoxia-hyperoxia on performance - and health-related outcomes in humans: a systematic review. Sports Med Open.

[35] Bayer U, Glazachev OS, Likar R (2017). Adaptation to intermittent hypoxia-hyperoxia improves cognitive performance and exercise tolerance in elderly. Adv Gerontol.

[36] Dudnik E, Zagaynaya E, Glazachev OS, Susta D (2018). Intermittent hypoxia-hyperoxia conditioning improves cardiorespiratory fitness in older comorbid cardiac outpatients without hematological changes: a randomized controlled trial. High Alt Med Biol.

[37] Glazachev O, Kopylov P, Susta D, Dudnik E, Zagaynaya E (2017). Adaptations following an intermittent hypoxia-hyperoxia training in coronary artery disease patients: a controlled study. Clin Cardiol.

[38] Serebrovskaya TV, Xi L (2016). Intermittent hypoxia training as non-pharmacologic therapy for cardiovascular diseases: Practical analysis on methods and equipment. Exp Biol Med (Maywood).

[39] Bestavashvili A, Glazachev O, Ibragimova S (2023). Impact of hypoxia-hyperoxia exposures on cardiometabolic risk factors and TMAO levels in patients with metabolic syndrome. Int J Mol Sci.

[40] Iliescu MG, Stanciu LE, Uzun AB (2024). Assessment of integrative therapeutic methods for improving the quality of life and functioning in cancer patients—a systematic review. J Clin Med.

[41] Irsay L, Ungur RA, Borda IM (2022). Safety of electrotherapy treatment in patients with knee osteoarthritis and cardiac diseases. Life (Basel).

[42] Lupu AA, Oprea D, Obada B (2023). Variation of serum serotonin values under specific peloido-therapy in patients with degenerative pathology of the lumbar spine. Balneo PRM Res J.

[43] Savulescu SE, Berteanu M, Filipescu I (2021). Repetitive peripheral magnetic stimulation (RPMs) in subjects with lumbar radiculopathy: an electromyography-guided prospective, randomized study. In Vivo.

[44] Mihai EE, Popescu MN, Beiu C, Gheorghe L, Berteanu M (2021). Tele-rehabilitation strategies for a patient with post-stroke spasticity: a powerful tool amid the COVID-19 pandemic. Cureus.

